# Correction: IL-22 inhibits ferroptosis and attenuates ischemia-reperfusion-induced acute kidney injury: Association with activation of the P62-Keap1-Nrf2 signaling pathway

**DOI:** 10.1371/journal.pone.0351066

**Published:** 2026-06-03

**Authors:** Lin Zhang, Feng Luo, Yalin Chai, Lijie Sun, Xuan Wang, Le Yin, Congjuan Luo

The Funding statement for this article is incorrect. The correct Funding statement is as follows: This work was supported by the Taishan Scholar Program of Shandong Province (No. tstp20230665), the Special Project of Benefiting People with Science and Technology of Qingdao (No. 26-1-5-smjk-4-nsh), and the National Natural Science Foundation of China (No. 82270724 and No. 82470722).
