# Supplementary material for: IL-22 inhibits ferroptosis and attenuates ischemia-reperfusion-induced acute kidney injury: Association with activation of the P62-Keap1-Nrf2 signaling pathway
Source: PLoS One. 2026 Feb 6;21(2):e0342335. doi: 10.1371/journal.pone.0342335 (PMC12880650; doi:10.1371/journal.pone.0342335)
Supplement: S1 Table — (DOCX) [file pone.0342335.s001.docx]

S1 Table. Primers Used in qRT-PCR.

| **Gene** | **Forward primer** | **Reverse primer** |
| --- | --- | --- |
| KIM-1 (mice) | ACTCCTGCAGACTGGAATGG | CAAAGCTCAGAGAGCCCATC |
| KIM-1 (human) | CTGCAGGGAGCAATAAGGAG | TCCAAAGGCCATCTGAAGAC |
| NGAL (mice) | ATGTCACCTCCATCCTGGTCAG | GCCACTTGCACATTGTAGCTCTG |
| NGAL (human) | GTGAGCACCAACTACAACCAGC | GTTCCGAAGTCAGCTCCTTGGT |
| Nrf2 (mice) | CGACAGCATGTCCCAGGATT | CTGGGTTCTGCTTGTTTCGC |
| Nrf2 (human) | TCAGCGACGGAAAGAGTATGA | CCACTGGTTTCTGACTGGATGT |
| GPX4 (mice) | GCACATGGTCTGCCTGGATA | GGGAAGGCCAGGATTCGTAA |
| GPX4 (human) | GAGGCAAGACCGAAGTAAACTAC | CCGAACTGGTTACACGGGAA |
| ACSL4 (mice) | CCACACTTATGGCCGCTGTT | GGGCGTCATAGCCTTTCTTG |
| ACSL4 (human) | TTGGGCATTCCTCCAAGTAG | CCTGCAGCCATAGGTAAAGC |
| SLC7A11 (mice) | GAGTGCCCGGATCCAGATTT | GGCAACCCCATTAGACTTGTG |
| SLC7A11(human) | ATGCAGTGGCAGTGACCTTT | GGCAACAAAGATCGGAACTG |
| SQSTM1(p62) (mice) | GCTCTTCGGAAGTCAGCAAACC | GCAGTTTCCCGACTCCATCTGT |
| SQSTM1(p62) (human) | TGTGTAGCGTCTGCGAGGGAAA | AGTGTCCGTGTTTCACCTTCCG |
| Keap1 (mice) | GGAGGAAGACTGAAGGAGGA | GGATGTCTGGTTGTCTGTGG |
| Keap1 (human) | CAACTTCGCTGAGCAGATTGGC | TGATGAGGGTCACCAGTTGGCA |
| GAPDH (mice) | GGTGAAGGTCGGTGTGAACG | CTCGCTCCTGGAAGATGGTG |
| GAPDH (human) | AGAAGGCTGGGGCTCATTTG | AGGGGCCATCCACAGTCTTC |
