# Supplementary figures and images for: IL-22 inhibits ferroptosis and attenuates ischemia-reperfusion-induced acute kidney injury: Association with activation of the P62-Keap1-Nrf2 signaling pathway
Source: PLoS One. 2026 Feb 6;21(2):e0342335. doi: 10.1371/journal.pone.0342335 (PMC12880650; doi:10.1371/journal.pone.0342335)

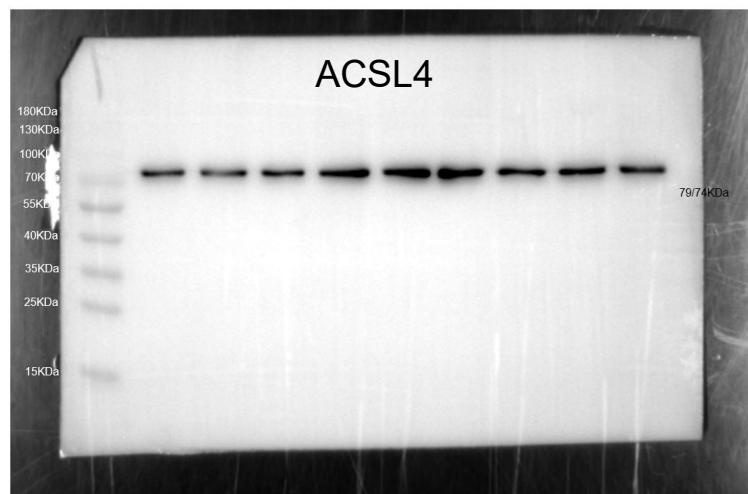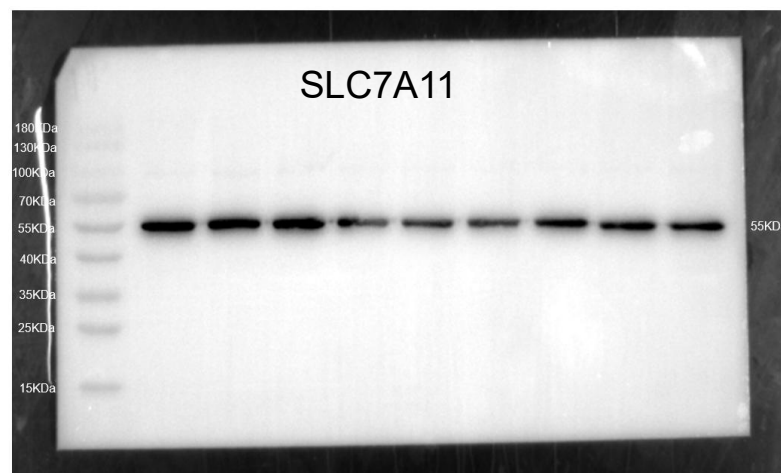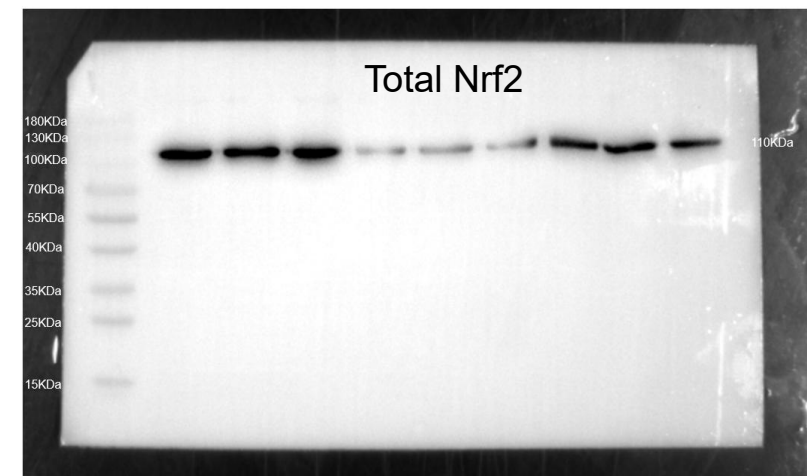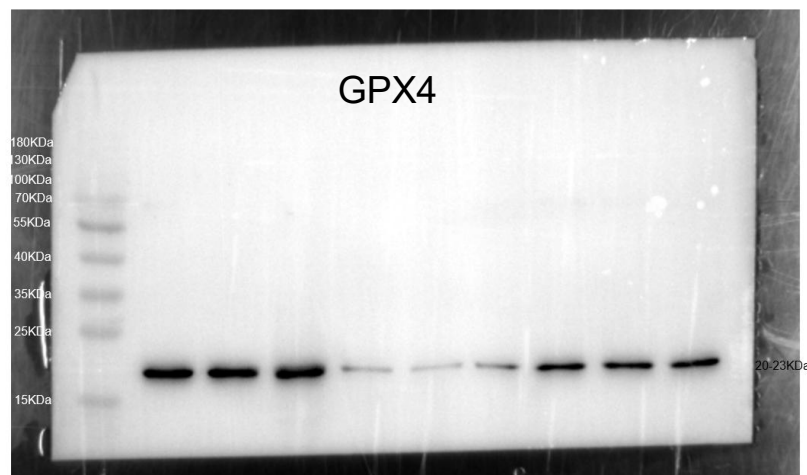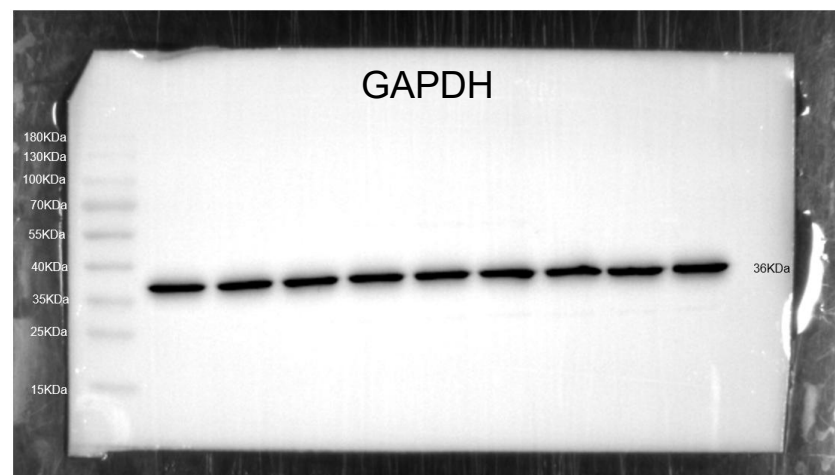

Fig 2

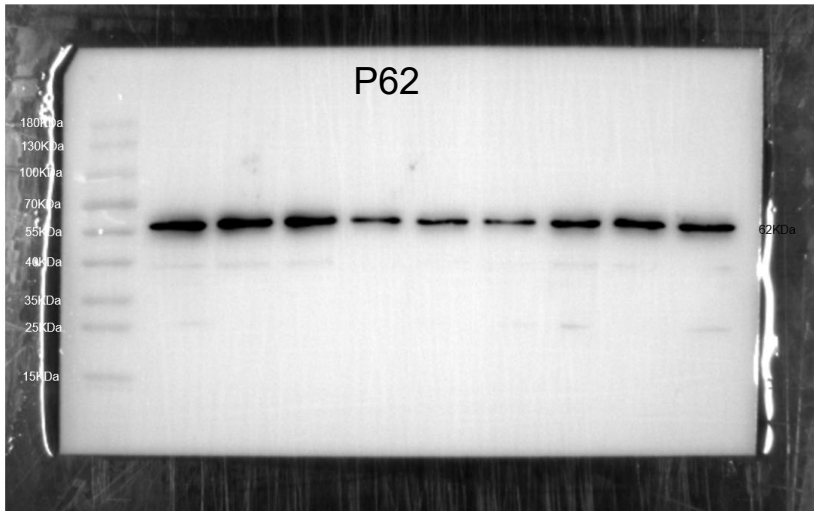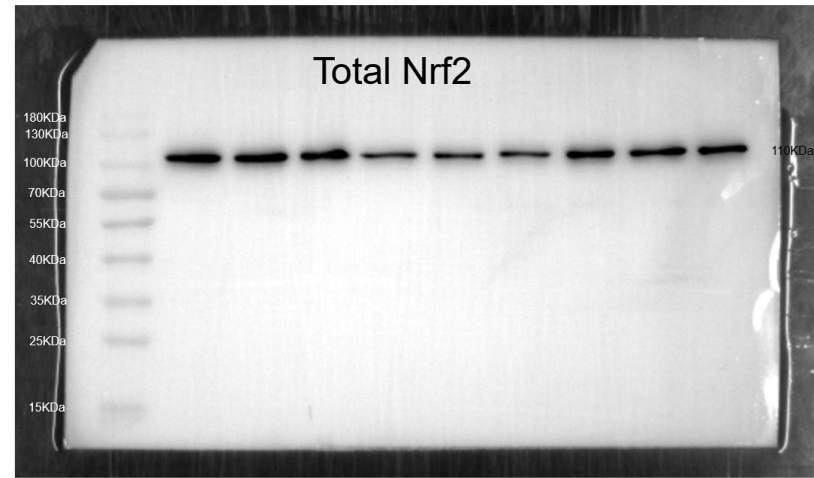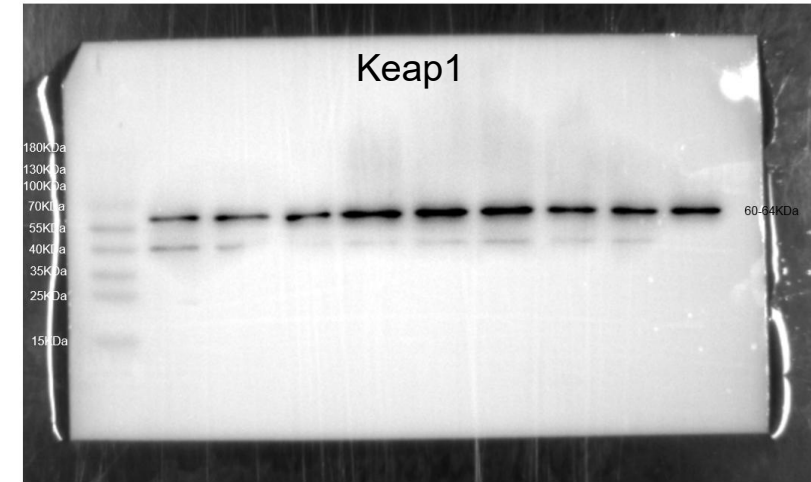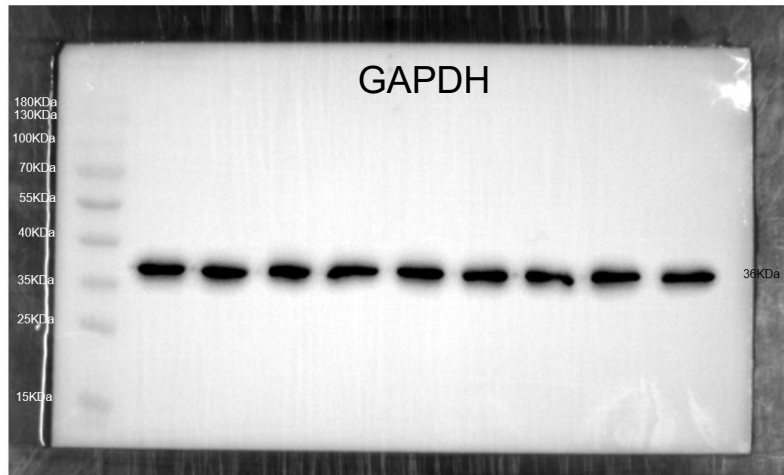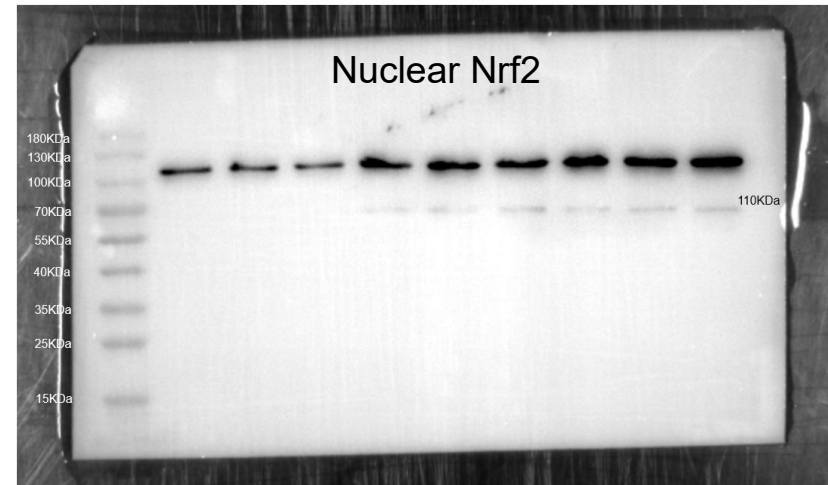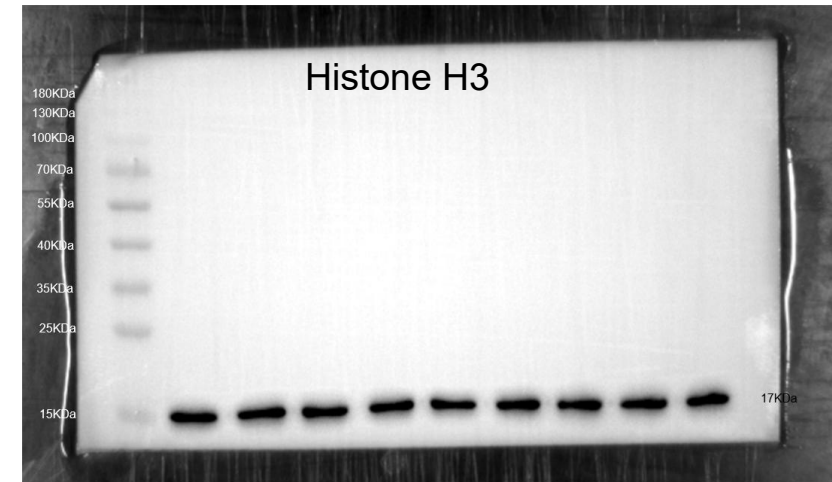

Fig 3

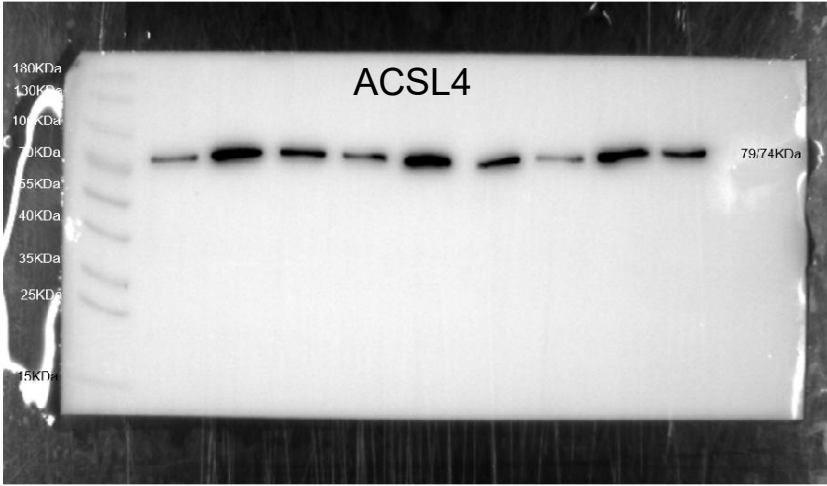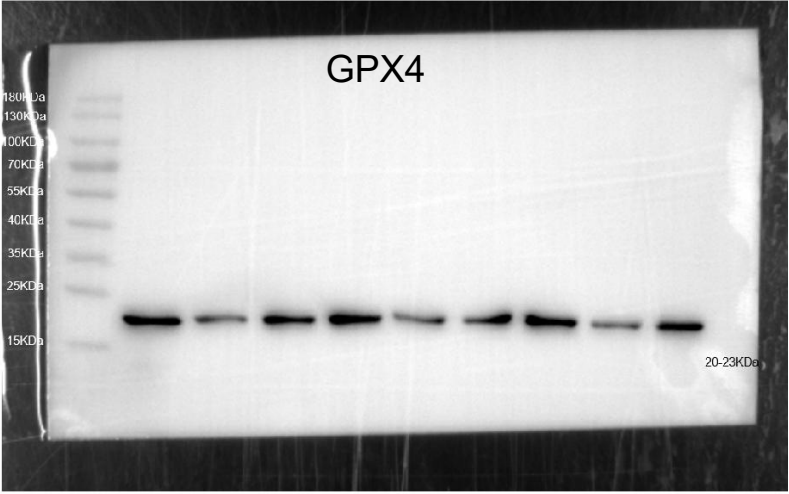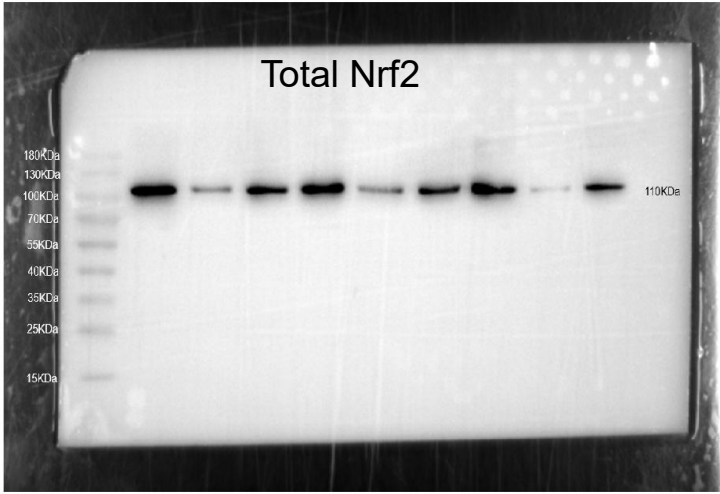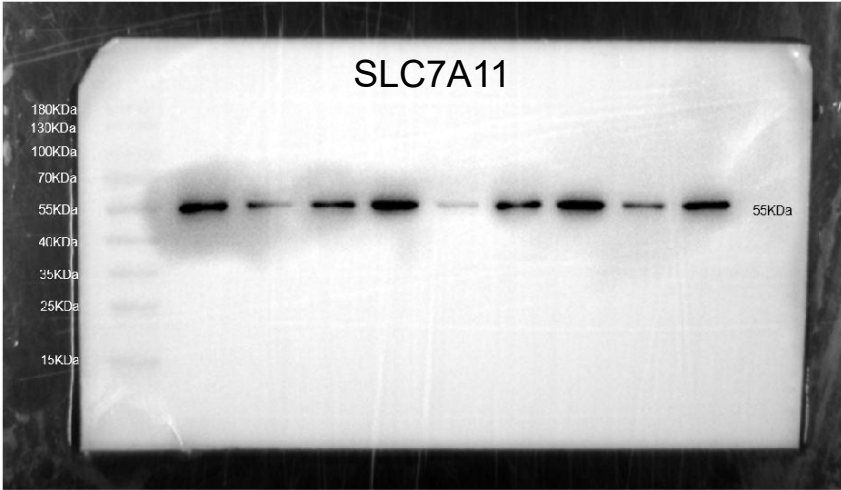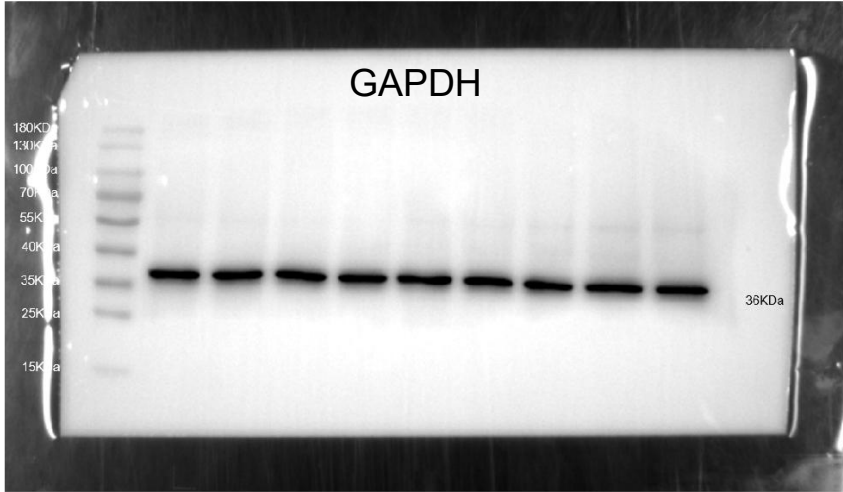

Fig 4

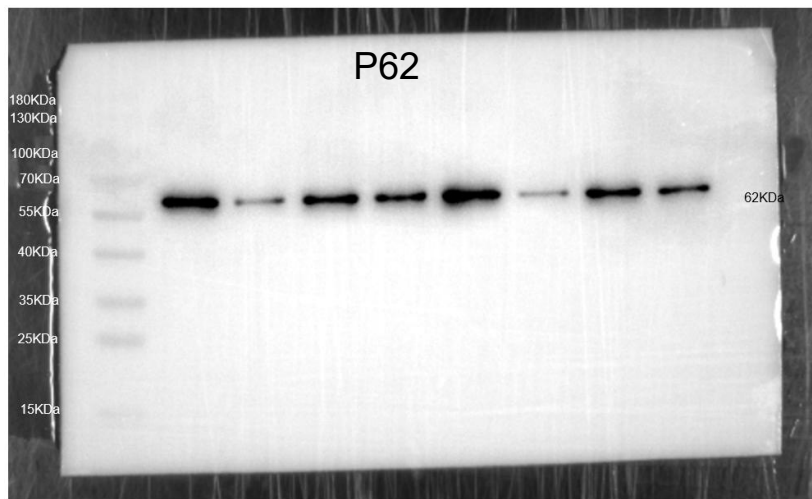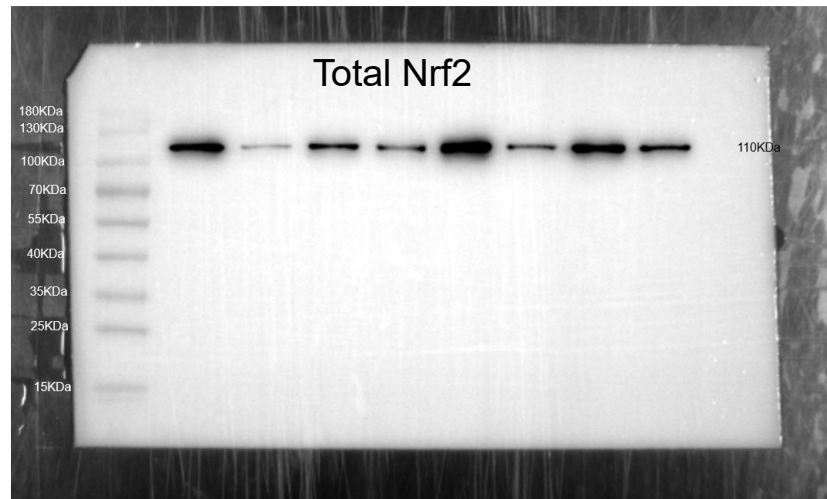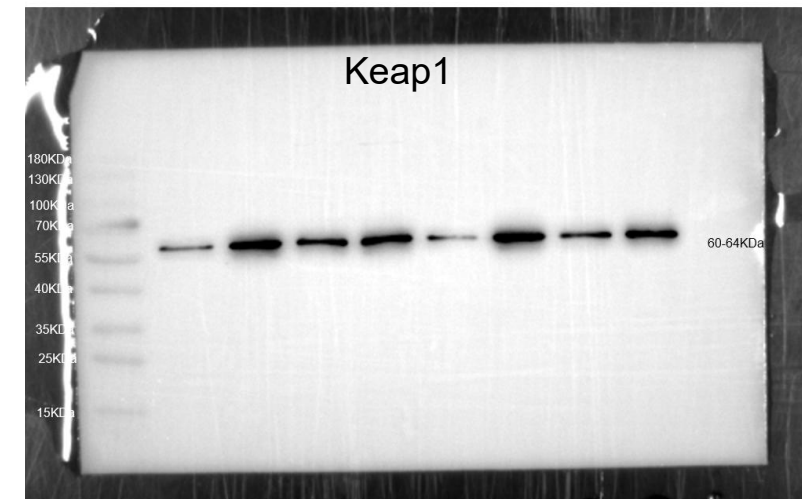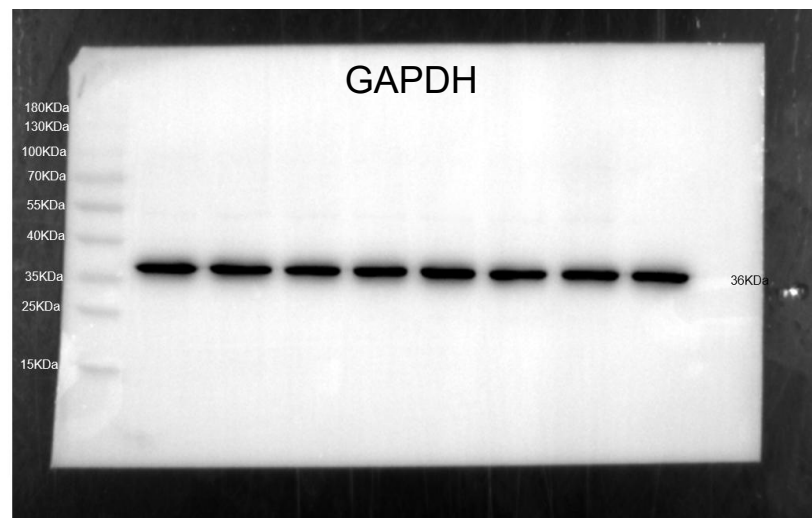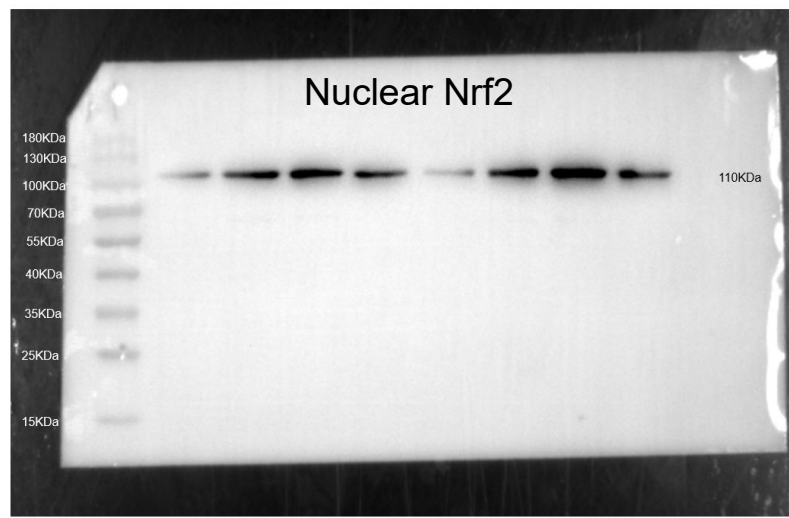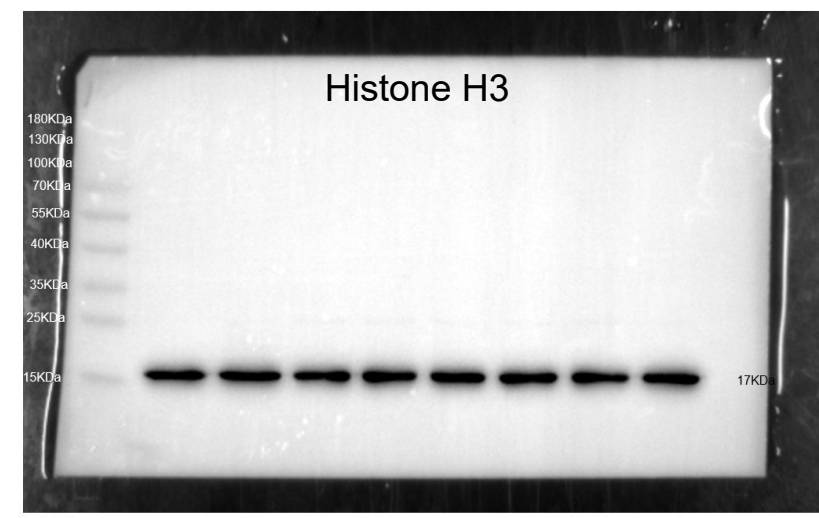

Fig 5

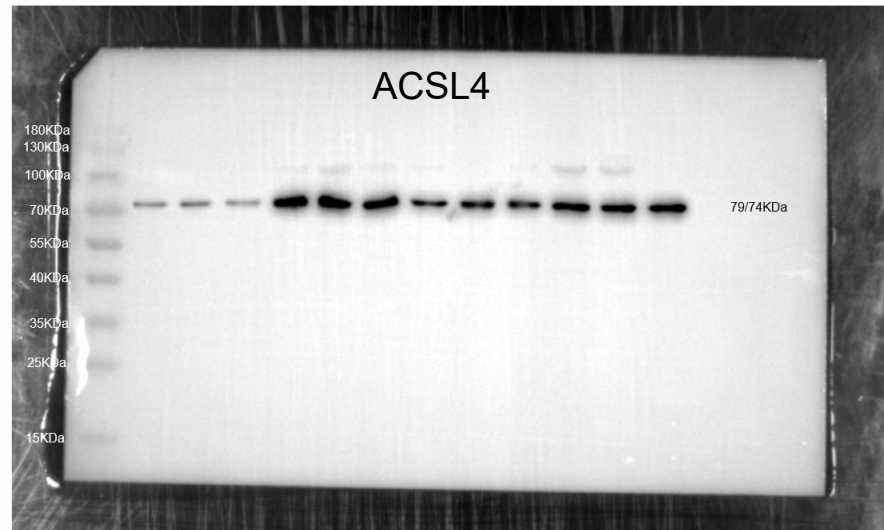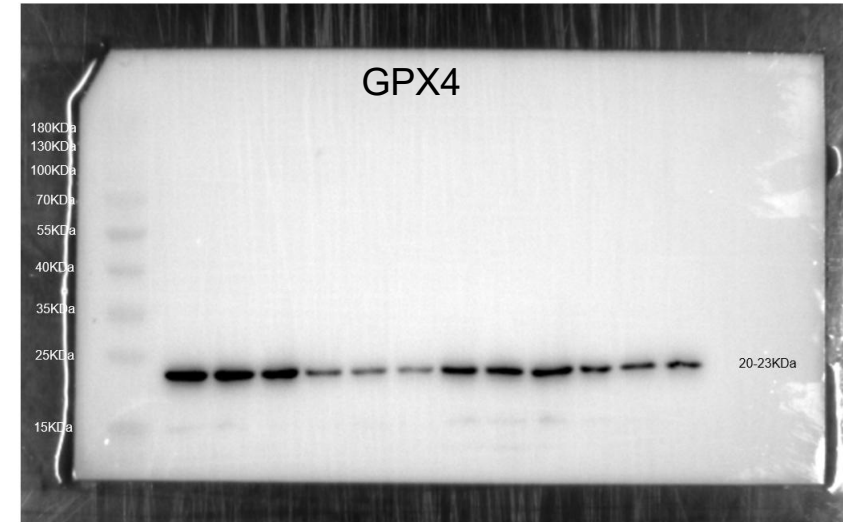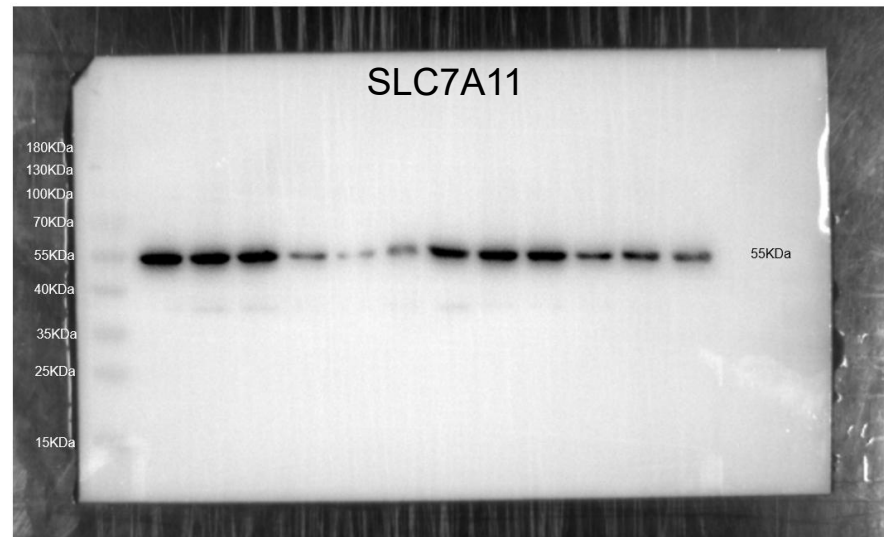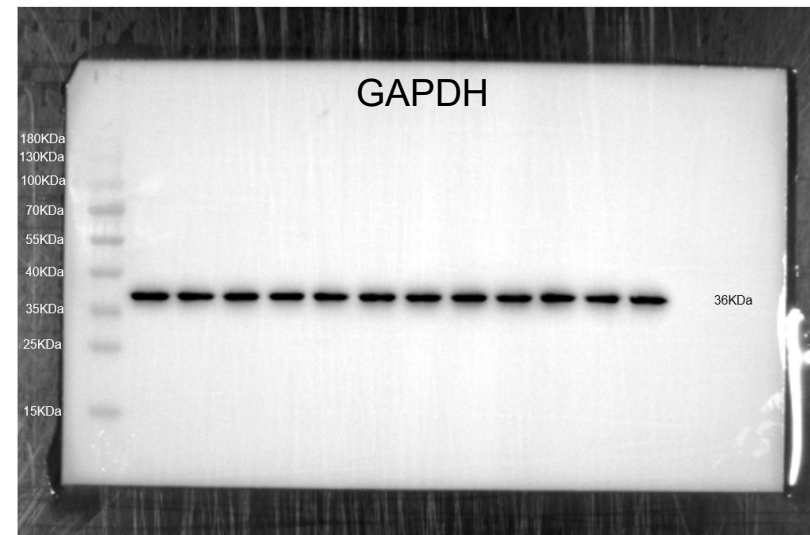

Fig 5

Supplement: S1 Raw Images — (PDF) [file pone.0342335.s002.pdf]
